# Media Source Characteristics Regarding Food Fraud Misinformation According to the Health Information National Trends Survey (HINTS) in China: Comparative Study

**DOI:** 10.2196/32302

**Published:** 2022-03-16

**Authors:** Angela Chang, Peter Johannes Schulz, Wen Jiao, Guoming Yu, Ya Yang

**Affiliations:** 1 Faculty of Social Sciences University of Macau Macao China; 2 Institute of Communication and Health Lugano University Lugano Switzerland; 3 School of Journalism and Communication Beijing Normal University Beijing China

**Keywords:** risk assessment, food rumor, risk management, conspiracy narratives, population health, food safety

## Abstract

**Background:**

Ongoing rumors and fake news regarding food fraud, adulteration, and contamination are highly visible. Health risk information circulating through media and interpersonal communication channels has made *health crisis* an important research agenda.

**Objective:**

This study explored the issue of food fraud and the effect of misinformation. Further, it assessed whether and how these issues have provided evidence-based interventions for food handlers and regulators to mitigate fraud misinformation.

**Methods:**

The Health Information National Trends Survey (HINTS) was adopted for a collaborative study in China, after which a cross-sectional survey with door-to-door interviews was performed. Participants from Beijing and Hefei were selected using multistage sampling of adults in May 2017. Based on 4 government surveillance reports on food rumors and safety incidents, a descriptive analysis, correlation analysis, and analysis of variance were performed on the data.

**Results:**

A total of 3090 results were gathered and analyzed. Among the respondents, 83.6% (2584/3090) heard at least one food rumor. Learning about food fraud was correlated with interpersonal connections (eg, doctors or health specialists) for accessing food health information. Overall, Chinese citizens with a higher level of interpersonal connection were more likely to be concerned about food incidents with a statistical difference (*P*<.001). Interpersonal connection was the most frequent communication source (698/1253, 55.7%), followed by traditional media (325/1253, 25.9%) and internet portals (144/1253, 11.5%). There was a significant relationship between media use and media category in Beijing (*P*<.001) and Hefei (*P*<.001). Overall, responses to food fraud and incident risks were lower in Beijing than in Hefei (*P*=.006). The respondents in Beijing were confronted more frequently by food rumors (range 346-1253) than those in Hefei (range 155-946). The urban dwellers in Beijing and their rural counterparts in Hefei also differed in terms of perceiving different levels of food risks from different media sources. The food rumor narratives that examined the conspiracy belief showed that social media played more important roles in influencing attitudes against misinformation for users in Hefei than in Beijing.

**Conclusions:**

This study shows that consumers have to be on guard against not only fake food, but also spreading fake information and rumors, as well as conspiracy beliefs involving fake food. This study focused on characterizing media sources, types of food fraud misinformation, and risk perceptions of food safety, which mix urgency and suspicion, and attempted to provide evidence-based interventions for risk management guidance, with the hypothesis of significant correlations between media types and sources, and consumer exposure and perception levels of food rumors and risks.

## Introduction

### Background

Food fraud has been practiced since ancient times but has become more sophisticated in recent years. The recent food supply chains have become complicated and accelerated in local markets. Government authorities may be able to trace some imported contaminated food items; however, they are smuggled from one country to another with switched packaging and altered production dates. Thus, the risk of food fraud has broadened to include the global population [1]. Food fraud that involves items, such as infant milk formula, olive oil, and Scotch whisky, has been examined for perception and attitude learning among consumers in first- and second-tier cities in China [2], and the conclusion indicated that food fraud represents a food hazard and poses a threat to the authenticity, quality, and reliability of food.

Investigating food fraud is an emerging research idea and is a newly defined area of food protection [1,3,4]. Food fraud is an intentional act for economic gain, whereas a food safety incident may be an unintentional act with unintentional harm [5]. Thus, studying food fraud and adulterants mainly focuses on food safety (eg, pesticide residue) and food defense with malicious intent to harm (eg, terrorism attack). Food fraud and adulteration are principally motivated by economic drive; however, food fraud–related public health risks are often riskier than traditional food safety threats [1,6]. This is because food contaminants are complicated risks, which may involve chemical, microbial, physical, and allergenic contaminants with food fraud conditions. This also explains why food handlers have a responsibility to ensure that the food they prepare is free from these contaminants and safe for the consumer [7].

In recent decades, health information has been particularly exposed to rumors, incidents, and fake news [6,8,9]. Until the recent 10 years, little was known about the access, sources, and trust levels of food fraud and related information, or factors that facilitate or hinder communication on a population-wide basis [10-12]. In order to help fill this gap, researchers have tried to employ conspiracy beliefs to explain health-related matters. Conspiracy beliefs adjoined to health information are reportedly on the rise and are beginning to receive significant attention (eg, water fluoridation, vaccines, cell phones, unhealthy diets, and alternative medicine) [9,13-15].

More recently, the outbreak of food incidents and rumors has provided evidence of how the spread of conspiracy thinking has reached beyond the narrow confines of individual or group believers [16-18]. A conspiracy belief works through emotional appeals and implausibility, which can be used as a rhetorical device to appeal to the emotions of a significant number of the public [19,20]. The conspiracy belief is significantly associated with health worries while considering variables such as demographics, ideology, and health perceptions [8,21]. However, it remains unclear whether the objective information of conspiracy beliefs matters.

China’s food safety rumors and concerns involve fraud, implicated foods, adulterants, contaminants, and abnormal conditions, as well as concerned food sources [4]. For many in China, the term genetically modified foods (GMFs) evokes negative reactions as it is usually connected with food safety concerns such as poisoned seeds or contaminated fields [22]. Vast food fraud and related studies have focused on GMFs for supposed damage [23-26].

While academia researchers, government regulators, and industry practitioners alike struggle with this evolving issue [4,27], the terms and definitions of food fraud should include deliberate substitution, dilution, counterfeiting, or misrepresentation of food, ingredients, or packaging, as well as false or misleading statements made about a food product. All these examples of food fraud can have a negative impact on the quality and safety aspects of food, some of which have resulted in serious illnesses and even fatalities, damaging trust in domestically produced and imported food.

### Media Exposure of Food Health Risk

Little is known about the sources and trust of food fraud information hindering communication on a population-wide basis. The Health Information National Trends Survey (HINTS) was developed to help fill this gap [28,29]. The HINTS reported that around 50% of American respondents preferred to go to their physicians for specific health information [30]; however, when asked where they actually went, up to 48.6% of respondents reported going online first.

People’s perceptions of health risks are initially determined by the scope and width of coverage from the media for the most part [8]. With the growing influence of social media in the public sphere, the communication of alternative health discourse often circulates in opposition to the mainstream or government-owned media [31-33]. Consequentially, the narratives supporting alternative health discourses have increasingly become the growing consensus for laymen rather than health professionals or policymakers [24,34,35].

In order to determine the extent of health conspiracism in public, social media might play an increasingly important role in active information-seeking behavior [15,36]. Although previous studies provide important insights for understanding active seeking behavior, they neglect the incidental seeking behavior of health information.

The steady growth of food safety rumors and incidents would increase citizens’ concerns, but media themselves inform, circulate, and even amplify the food safety risk perceived. In fact, tensions arise between medical science protecting the collective well-being and the emotive amplification of groups concerned with individual health. Such exchanges have developed in food health and safety discourses, with online forums working as echo chambers [37].

### Goal of This Study

The narratives of scandals and rumors might correlate with media sources and impact actual health information seeking behaviors. Thus, this study focused on characterizing media sources, the types of food fraud misinformation, and the risk perception of food safety, which mixes urgency and suspicion.

The information regarding food risks and safety is becoming more relevant [7]. If information is viewed in a more comprehensive and complex way, the resulting importance of the topic would encourage clarification of the types of food fraud and consequentially which conspiracy beliefs are salient and whether the public has prescribed to these beliefs.

### Research Questions

The abovementioned conspiracy beliefs are associated with the behavior of seeking information that impacts food fraud. Thus, the risk perceptions of food could be different for passive information seeking behavior. Concomitantly, demographic variables are significantly related to the concerns of food fraud and adulteration type [7,38]. Thus, the following 5 research questions were raised: (1) What types of food fraud–related incidents and rumors are spreading fear in China, regarding food safety concerns? (2) Are the conspiracy beliefs associated with demographic variables, such as income, age, gender, and education? (3) Is learning about food fraud correlated with media type and source? (4) Are citizens with more social media exposure more likely to be concerned about food rumors and scandals? (5) Is there any difference in the perception level of food risk between urban dwellers and their rural counterparts?

## Methods

### Data Collection

The Health Information National Trends Survey in China (HINTS-China) project extends the established HINTS from the United States [11,39-41]. The questionnaire was built upon the research framework of the HINTS by using identical core questions, as well as adding unique food fraud health misinformation and concerns, and communication characteristics for the Chinese public. It is a cooperative project supported by Beijing Normal University, George Mason University, the National Cancer Institute in the United States, the Chinese Food and Drug Administration, and the Chinese Health Media Group. The HINTS-China was approved by the institutional review board of the School of Journalism and Communication, Beijing Normal University in 2017.

Data were collected in 2017 from Beijing, the capital city of the People’s Republic of China, and Hefei, a second-tier and capital city in Anhui Province. Considering the different developments in political and economic applications, this study compared the commonalities and differences of the 2 locations to learn the impacts of food rumors and incidents on consumers [11,39,42,43]. With respect to urban and rural respondents, it aimed to provide a picture of the information communication strategy and risk assessment.

A multistage stratified sampling method was employed, and the procedure included training interviewers for door-to-door interviews between March to May in 2017. The interviews took place from May 9 to 24, 2017. One member aged 18 years or above from each household was requested to take part. Respondents who took part in the survey gave their written consent [43].

### Conspiracy Narratives of Food Misinformation

The spread of malicious or accidental rumors online, particularly regarding food in real-world emergencies, can have harmful effects on the society. Thus, 8 highly publicized narratives of food risks were officially identified as rumors before implementing the HINTS-China. The veracity of food rumors comprised a range of food additives, fraudulent activities, adulterant substances, GMFs, and food contamination. For example, suspected rumors of food narratives were as follows: “seaweed (nori) is made out of black plastic,” “big and sweet strawberries are with excessive swelling agent,” and “seedless grapes are covered with contraceptives.” A list of food fraud narratives with English translations is displayed in Textbox S1 in Multimedia Appendix 1.

### Health Risks of Food Fraud

Eleven heated food safety incidents in 2016 were exposed. These were included in the HINTS-China to measure consumers’ awareness and perception of health risks. The veracity of negative food exposure comprised the food-origin story, food additives, adulterants, authenticity, crime, contamination, integrity, quality, and food security. For instance, the narratives regarding food safety were as follows: “seafood from radiated areas in Japan were smuggled into China,” “more than 100 tons of expired milk powder from New Zealand was illegally repackaged and sold in Shanghai,” “A Vietnam yogurt brand with three-no yogurt (low-quality product with no manufacturer name, no production site, and no production hygiene license code) entered the market in many cities in mainland China, and the product was seized by the China Food & Drug Administration (CFDA),” and “A food ordering platform was accused of partnering with an unlicensed restaurant with poor sanitary conditions by a government-owned television program.” A list of health risks of food incidents with safety concerns is shown in Textbox S2 in Multimedia Appendix 1.

### Detection and Verification Mechanism

Many food risk concerns and incidents in China were initially reported by central official surveillance and then by local media. The state-owned news media detected food fraud and adulteration in news content and also delivered information concerning food authenticity and integrity. The salient features of rumors and food safety were identified from the following 2 official sources: People’s Daily Online (人民網) and Xinhuanet (新華網) [4,44]. People’s Daily is supported by the Central Committee of the Chinese Communist Party, while the XinHua Press Agency is an official agency and is the largest news agency in the world.

Two more sources joined the verification mechanism of food rumors and incidents, namely, Chinese Health Daily News (中国健康报) and CNPharm.com (中国食品药品网). Online media provide professional and reliable information under the supervision of the National Health Commission. It has been confirmed that all of the 8 popular rumors in Textbox S1 in Multimedia Appendix 1 are false, while the narratives of the 11 food scandals in Textbox S2 in Multimedia Appendix 1 reflect the facts in 2016, though 2 of them are not accurate.

### Measurements

We measured the conspiracy narratives of food fraud, rumors, incidents, safety issues, characteristics of media sources, and scale descriptions. In particular, the measurements of the narratives of food rumors and incidents were to learn whether the media sources that people searched for on hearing about a statement were trustworthy.

The established measures included a dichotomous answer of yes/no for having heard about each food rumor or incident. It was followed by a 5-point Likert scale for the measurement of the degree of trust, ranging from 1 (completely trust) to 5 (completely distrust), with a higher score indicating a higher distrust of the food rumor and a lower score indicating a lower distrust of the food rumor. The socioeconomic information measured included gender, age, ethnic group, occupation, marital status, highest level of education, and monthly income.

Regarding the media source, the HINTS-China measured the media use behavior regarding seeking general information and health information. The first question focused on 12 media characteristics, either general purpose or health/medical-related information in the past 12 months. A dichotomous question checked people’s active information seeking process regarding health/medicine. Moreover, a total of 25 media sources were provided to learn their impact on food rumors and incidents. In order to merge the media sources, the following 5 categories were established for analysis: (1) interpersonal media, (2) public organizations, (3) traditional media, (4) internet portals, and (5) social media (Multimedia Appendix 2). One media option was excluded from the analysis because it appeared that none or very few people had accessed it (less than 1%).

Descriptive and correlation analyses were performed using SPSS 24 (IBM Corp). For any accessed media that connected with food misinformation and food safety issues, analysis of variance (ANOVA) was employed, which determined whether the influence of media use mediated the conspiracy narratives of food rumors and incidents.

## Results

### Descriptive Analysis

As of May 2017, we conducted the survey and data collection, which was funded by CFDA and Chinese Health Media Group in January 2017, and approved by the institutional review board at the School of Journalism and Communication, Beijing Normal University in March 2017. After 16 dates of data collection (May 9 to 24), 3090 samples were collected, and the number of valid questionnaires was 2584, including 1462 in Beijing and 1122 in Hefei, after list-wise deletion of missing values. The preliminary description was made in August 2017, and further data analysis was performed in May 2020.

The response rate was 42.65% for Beijing and 57.35% for Hefei (out of 3090 participants). The dominant ethnic group was Han Chinese, ranging from 98.0% (1433/1462) in Beijing to 99.5% (1116/1122) in Hefei. Respondents’ ages ranged from 18 to 60 years (mean 38.02 years, SD 11.16) in Beijing, but the respondents were younger on average in Hefei (mean 32.26 years, SD 11.23). In Beijing, respondents were predominantly female (920/1462, 62.9%), married or in cohabitation (1199/1462, 82.0%), and high school educated (553/1462, 37.8%). In comparison, Hefei’s respondents were predominantly female (677/1122, 60.3%), married or in cohabitation (696/1122, 62.0%), and college educated with a bachelor’s degree (351/1122, 31.3%). Table 1 displays the sociodemographic findings of respondents in Beijing and Hefei in 2017.

**Table 1:** Sociodemographic findings of respondents in Beijing and Hefei in 2017.

Characteristic			Beijing (N=1462), n (%)		Hefei (N=1122), n (%)
**Gender**					
	Male	542 (37.1)		445 (39.7)	
	Female	920 (62.9)		677 (60.3)	
**Occupation**					
	General staff in the organization	342 (23.4)		82 (7.3)	
	Worker or self-employed	307 (21.0)		289 (25.8)	
	Business service staff	229 (15.7)		45 (4.0)	
	Retiree or unemployed	267 (18.3)		151 (13.5)	
	Government administrator	15 (1.0)		29 (2.6)	
	Professional technician	95 (6.5)		101 (9.0)	
	Enterprise administrator	88 (6.0)		116 (10.3)	
	Education sector	37 (2.5)		54 (4.8)	
	Student	31 (2.1)		207 (18.4)	
	Private entrepreneur	24 (1.6)		36 (3.2)	
	Agricultural laborer	27 (1.8)		12 (1.1)	
**Marital status**					
	Married or cohabiting	1199 (82.0)		696 (62.0)	
	Single, never married	243 (16.6)		399 (35.6)	
**Education level**					
	Primary school or below	28 (1.9)		30 (2.7)	
	Junior middle school	288 (19.7)		148 (13.2)	
	High school	553 (37.8)		200 (17.8)	
	Junior college	362 (24.8)		308 (27.5)	
	Bachelor’s degree	219 (15.0)		351 (31.3)	
	Master’s degree or above	12 (0.8)		85 (7.6)	
**Individual income (RMB)^a^**					
	No income	76 (5.2)		243 (21.7)	
	Below 1000	11 (0.8)		28 (2.5)	
	1000-2499	194 (13.3)		203 (18.1)	
	2500-4999	719 (49.2)		378 (33.7)	
	5000-9999	413 (28.2)		210 (18.7)	
	10,000 or above	49 (3.4)		60 (5.3)	

^a^1 RMB = 0.158 USD.

Overall, interpersonal communication was observed to be the most favorable first choice of media use for accessing food risk information (n=1466), followed by internet portals (n=464) and traditional media (n=425). A chi-square test of independence showed that there was a significant relationship between media use and media category in Beijing (χ^2^_8_=68.223; *P*<.001) and in Hefei (χ^2^_8_=78.529; *P*<.001). The rank of media preference greatly impacts food information outcomes. The additional insight shows that merging use of media sources mimics the way we now consume content across devices and platforms. Table 2 outlines the ranking of media sources for accessing food information in Beijing and Hefei.

**Table 2:** Ranking of media sources for accessing food risk information in Beijing and Hefei.

Media source	Choice of media in Beijing					Choice of media in Hefei			
	1st (n=572), n (%)	2nd (n=571), n (%)	3rd (n=572), n (%)	Subtotal, n	1st (n=426), n (%)		2nd (n=373), n (%)	3rd (n=410), n (%)	Subtotal, n
Interpersonal communication	385 (39.3)	331 (33.8)	263 (26.9)	979	209 (42.9)		155 (31.8)	123 (25.3)	487
Internet portals	69 (29.7)	65 (28.0)	98 (42.2)	232	98 (42.2)		72 (31.0)	62 (26.7)	232
Traditional media	75 (25.5)	111 (37.8)	108 (36.7)	294	34 (26.0)		24 (18.3)	73 (55.7)	131
Public organizations	34 (22.7)	47 (31.3)	69 (46.0)	150	63 (24.7)		90 (35.3)	102 (40.0)	255
Social media	9 (15.0)	17 (28.3)	34 (56.7)	60	22 (21.2)		32 (30.8)	50 (48.1)	104

Specifically, in Beijing, seeking doctors’ or health specialists’ advice concerning food risk information was presented most frequently (264/362, 72.9%), followed by a family member (197/324, 60.8%) and television (57/162, 35.2%). However, in Hefei, seeking doctors’ or health specialists’ advice remained the first option (112/151, 74.2%), followed by a family member (85/169, 50.3%) and friends and colleagues (69/167, 41.3%). Multimedia Appendix 3 displays the preferred media for accessing food risk and related information in Beijing and Hefei.

An average of 83.6% (n=2584) of Chinese respondents heard at least one food rumor, which included 95.7% (n=1462) from Beijing and 71.8% (n=1122) from Hefei. The respondents in Beijing were confronted more frequently by food rumors (range 346-1253) than those in Hefei (range 155-946). Interpersonal connection was the most frequent communication source (698/1253, 55.7%), followed by traditional media (325/1253, 25.9%) and internet portals (144/1253, 11.5%).

The K-means cluster identified 2 groups of users for learning narratives regarding food rumors. To be specific, in the high conspiracy belief group in Beijing, the category of interpersonal connection had the highest frequency of learning food rumor (130/275, 47.3%), followed by traditional media (72/275, 26.2%) and internet portals (49/275, 17.8%). A chi-square test of independence examined the conspiracy belief and media sources, and showed statistical significance (χ^2^_5_=20.08 [n=1253]; *P*=.001). A similar trend was also observed for the respondents in Hefei. For high conspiracy belief respondents, the category of interpersonal communication was the most frequent media source (182/346, 52.6%), followed by traditional media (113/346, 32.7%) and internet portals (31/346, 9.0%). However, there was no statistical significance for the high and low conspiracy beliefs in Hefei. Table 3 displays the crosstab comparison of conspiracy beliefs with access to the different categories of media sources in Beijing and Hefei in 2017.

**Table 3:** Crosstab comparison of conspiracy beliefs with access to different media sources in Beijing and Hefei.

Media source	Beijing, n (%)			Hefei, n (%)		
	Total (N=1253)	Low conspiracy belief (n=978)	High conspiracy belief (n=275)	Total (N=946)	Low conspiracy belief (n=600)	High conspiracy belief (n=346)
Interpersonal media	698 (55.7)	568 (58.1)	130 (47.3)	534 (56.4)	352 (58.7)	182 (52.6)
Public organizations	12 (1.0)	10 (1.0)	2 (0.7)	8 (0.8)	5 (0.8)	3 (0.9)
Traditional media	325 (25.9)	253 (25.9)	72 (26.2)	280 (29.6)	167 (27.8)	113 (32.7)
Internet portals	144 (11.5)	95 (9.7)	49 (17.8)	82 (8.7)	51 (8.5)	31 (9.0)
Social media	72 (5.7)	51 (5.2)	21 (7.6)	32 (3.4)	16 (2.7)	16 (4.6)
Others	2 (0.2)	1 (0.1)	1 (0.4)	10 (1.1)	9 (1.5)	1 (0.3)

The top publicized food rumor in Beijing was counterfeit seaweed (mean score 2.98, SD 1.05), followed by GMF involving chicken with 6 wings (mean score 2.69, SD 0.83) and seedless grapes with contraceptives (mean score 2.62, SD 0.96). In comparison, the most highly publicized rumor in Hefei was counterfeit seaweed (mean score 2.86, SD 0.98), followed by seedless grapes with contraceptives (mean score 2.65, SD 0.86) and GMF involving chicken with 6 wings (mean score 2.62, SD 0.90). Table 4 outlines the analysis of food fraud with rumors and its correlation with consumer conspiracy beliefs in Beijing and Hefei.

**Table 4:** Correlation analysis of food fraud with rumors and consumer trust in Beijing and Hefei.

Food rumors and related terms	Beijing (n=1462), mean score (SD)	Hefei (n=1122), mean score (SD)	Correlation
1. Food counterfeit: seaweed	2.98 (1.05)	2.86 (0.98)	0.044
2. Addictive: strawberry	2.12 (0.82)	2.49 (0.79)	−0.012
3. Safety: microwaved food	2.45 (0.93)	2.60 (0.84)	−0.050
4. Implicated food: instant noodles	1.98 (0.84)	2.28 (0.84)	−0.103^a^
5. Contaminated: crayfish	2.45 (1.03)	2.47 (0.96)	−0.013
6. Safety: hookworm in pork	2.32 (0.97)	2.62 (0.99)	−0.076
7. GMF^b^: Six-wing chicken	2.69 (0.83)	2.62 (0.90)	−0.046
8. Authenticity: seedless grapes with contraceptives	2.62 (0.96)	2.65 (0.86)	−0.004

^a^*P*<.01.

^b^GMF: genetically modified food.

The K-means cluster identified 2 groups of distrust levels for learning narratives regarding food rumors. All of the narratives regarding food fraud with rumors showed a statistical significance between users with a low level of distrust and their counterparts with a high level of distrust in Beijing and Hefei.

Specifically, more users with a higher score indicated a high distrust of food rumors, such as “counterfeit seaweed” (202/638, 31.7%) and “implicated food of instant noodles” (275/1253, 21.9%) in Beijing. In comparison, a low distrust of food rumors, such as the implicated food narrative “instant noodles are junk food” (600/946, 63.4%) and another narrative “crayfish are genetically modified crops which deals with corpses and grows in unsanitary water with exceeded heavy metals” (266/296, 89.9%), was prevalent in Hefei. Interestingly, much more users in Hefei distrusted the rumor narrative “big and sweet strawberries are with excessive swelling agent” (235/547, 43.0%) than their counterparts in Beijing (27/800, 3.5%). Another rumor regarding the use of genetically modified chicken with 6 wings by KFC also had contrasting trust levels in the 2 cities, with lower distrust in Beijing (94/794, 11.8%) and higher distrust in Hefei (182/449, 40.5%). Table 5 presents the findings of univariate ANOVA comparing the food items in frauds and rumors perceived by users with different levels of distrust.

**Table 5:** Comparison and analysis of distrust levels of rumors among users in Beijing and Hefei.

Food fraud and related terms	Beijing users					Hefei users				
	Low, n (%)	High, n (%)	*F* test (*df*)	*P* value	Low, n (%)		High, n (%)	*F* test (*df*)	*P* value	
1. Food counterfeit: seaweed	436 (68.3)	202 (31.7)	12277.62 (725)	<.001	253 (81.6)		57 (18.4)	259.67 (308)	<.001	
2. Additive: strawberry	773 (96.5)	27 (3.5)	214.67 (801)	<.001	312 (57.0)		235 (43.0)	1828.17 (545)	<.001	
3. Safety: microwaved food	646 (90.2)	70 (9.8)	533.26 (714)	<.001	279 (88.9)		35 (11.1)	216.77 (312)	<.001	
4. Implicated food/safety: instant noodles	978 (78.1)	275 (21.9)	2392.72 (1251)	<.001	600 (63.4)		346 (36.6)	2244.68 (944)	<.001	
5. Contamination/quality/genetically modified food: crayfish	381 (87.4)	55 (12.6)	374.62 (434)	<.001	266 (89.9)		30 (10.1)	207.05 (294)	<.001	
6. Safety/authenticity: pork hookworm	426 (88.6)	55 (11.4)	383.50 (479)	<.001	216 (85.4)		37 (14.6)	20.51 (10)	<.001	
7. KFC GMF^a^: Six-wing chicken	700 (88.2)	94 (11.8)	616.90 (792)	<.001	267 (59.5)		182 (40.5)	1047.60 (447)	<.001	
8. Authenticity: grapes with contraceptives	302 (87.3)	44 (12.7)	274,60 (344)	<.001	138 (89.0)		17 (11.0)	112.21 (153)	<.001	

^a^GMF: genetically modified food.

The terminology within the domain of food fraud and rumors included the following: food authenticity, food additive, food safety, food contamination, implicated food, and questionable GMF sources. The most significant misinformation of food fraud in rumors included “seaweed is made out of plastic” (χ^2^_4_=29.26; *P*<.001), “instant noodles are junk food” (χ^2^_4_=20.08; *P*<.001), “crayfish are GM crops” (χ^2^_4_=29.87; *P*<.001), “hookworm in pork” (χ^2^_4_=40.18; *P*<.001), and “seedless grapes were sprayed with contraceptives” (χ^2^_4_=19.95; *P*<.001) in Beijing. In comparison, only 1 issue regarding the rumor “seaweed is made of plastic” with different levels of distrust attitudes showed statistical significance in Hefei (χ^2^_4_=17.66; *P*=.003). Multimedia Appendix 4 shows the comparative analysis of the groups (low and high distrust) for learning about food rumors by accessing different media sources in Beijing and Hefei.

The top publicized food incidents perceived in Beijing were branded contaminated yogurt from Vietnam (mean score 4.08, SD 1.27), followed by suspected tainted tap water (mean score 4.02, SD 1.29) and expired milk powder from New Zealand (mean score 3.99, SD 1.31). In comparison, the most highly publicized news exposure in Hefei was the use of poppy shells in a chain restaurant in Beijing (mean score 4.12, SD 1.09), followed by a tainted sesame product (mean score 4.02, SD 1.12) and branded contaminated yogurt from Vietnam (mean score 4.01, SD 1.16). Table 6 outlines the analysis of food fraud items in scandals correlated with measuring consumer conspiracy beliefs between Beijing and Hefei.

**Table 6:** Food items and incident types involving consumer trust in Beijing and Hefei.

Food incident and related terms	Beijing (n=1462), mean score (SD)	Hefei (n=1122), mean score (SD)	Correlation
1. Implicated foods/abnormal conditions: fish out of shelves	3.67 (1.34)	4.03 (1.04)	−0.033
2. Food quality/security: delivery	3.19 (1.46)	3.53 (1.22)	−0.073^a^
3. Food adulterant: beef with duck meat	3.73 (1.37)	3.96 (1.13)	−0.060^a^
4. Food crime: poppy shells	3.74 (1.40)	4.12 (1.09)	−0.080^b^
5. Food authenticity: fake milk powder	3.86 (1.34)	3.49 (1.29)	−0.040
6. Food contaminant/security: radiation in seafood	3.65 (1.39)	3.47 (1.31)	−0.040
7. Food quality: expired dairy products	3.99 (1.31)	3.95 (1.13)	−0.049
8. Food contaminant: tainted sesame	3.86 (1.35)	4.02 (1.12)	−0.046
9. Food quality: frozen meat	3.90 (1.37)	3.81 (1.24)	−0.068^a^
10. Food quality/contaminant: yogurt	4.08 (1.27)	4.01 (1.16)	−0.019
11. Food contaminant: suspected tainted water	4.02 (1.29)	3.95 (1.16)	−0.022
Subtotal	3.79 (1.00)	3.85 (0.80)	−0.082^b^

^a^*P*<.05.

^b^*P*<.01.

The food fraud incidents and related terminology included food authenticity, food additive, food adulterant, food contamination, implicated food, food quality, and concerned sources with GMFs. The K-means cluster also identified 2 groups with distrust levels (low vs high) against negative food reports. All of the narratives regarding food fraud with scandals showed a significant difference between low-level distrust users and their high-level distrust counterparts in Beijing and Hefei.

Overall, responses to the food fraud and incident risks were lower in Beijing than in Hefei (*r*=−0.082; *P*=.006). The following 4 scandals reflected different perceptions between dwellers in Beijing and Hefei: food hygiene on an online food ordering platform (*r*=−0.073; *P*=.014), food adulteration by mixing beef with duck meat (*r*=−0.060; *P*=.046), food crime by using poppy shells (*r*=−0.080; *P*=.007), and food quality in problematic frozen meat (*r*=−0.068; *P*=.02). Multimedia Appendix 5 shows the comparative analysis of the groups (low and high distrust) for accessing media sources in learning about food in fraud and negative reports in Beijing and Hefei.

## Discussion

### Principal Findings

The main findings of this study were that around 73.6% (out of 2584) of Chinese respondents preferred to go to their physicians for quarrying food health information first; however, when asked where they actually went and got access to food rumors, up to 36.6% (out of 1462) of Beijing respondents and 55.6% (out of 1122) of Hefei respondents reported going online first. Interpersonal connection had the highest frequency among communication sources (698/1253, 55.7%). There was a significant relationship between media use and media category in Beijing (*P*<.001) and Hefei (*P*<.001). Overall, responses to the food fraud and incident risks were lower in Beijing than in Hefei (*P*=.006). The urban dwellers in Beijing and their rural counterparts in Hefei also differed in terms of perceiving different levels of food risk from different media sources.

Foods or raw ingredients most likely to be targeted for adulteration or fraud are those of high economical value to the diet, which are subject to the vagaries of weather during their growth, harvest, storage, or transport. Food quality and food safety incidents involving criminal conduct are considered as intentional acts, and there is zero tolerance from the food regulators in China. Consumers have to be on guard against not only fake food, but also spreading fake information and rumors about food.

Most Chinese respondents were principally confronted with food rumors through interpersonal connections, followed by traditional media and internet portals. Believing in conspiracies supports alternative views to construct radical beliefs in social uncertainty, and laypeople are now creating new articulations of discourse in the public sphere. This is because of their innovative and often subversive language. Despite the characterization of conspiracy beliefs as paranoid by some in public discourse, they remain robust sources of skepticism regarding important public health recommendations able to prevent the spread of rumors.

For example, a food rumor regarding seafood products being exposed to radiation has been highly publicized. Many countries, including China, have banned seafood imports from Fukushima and other Japanese prefectures due to contamination of surrounding waters [45]. Further, the massive 2011 Fukushima nuclear disaster led to dramatic price drops in Japanese seafood. Consumers were warned that radiation continues to negatively impact marine life in the vicinity of Fukushima. Thus, many consumers believe that seafood products have high levels of radiation that can cause irreversible damage to human cells. Although 10 years have passed since the disaster, the appetite of many consumers of Japanese seafood has remained low.

In our analysis of the prevalence of food incident–related conspiracy beliefs, we found that acceptance of those beliefs highlighted the narratives of seafood contaminants for Beijing respondents (mean score 3.65, SD 1.39) and the narratives of food crime in adding poppy shells for Hefei respondents (mean score 4.12, SD 1.09). Overall, the level of conspiracy belief acceptance grew over the issues of food quality and contamination in Beijing, while the level of acceptance was increasing over the issues of food crime and contamination in Hefei. As with many conspiracies, these beliefs can be accepted by the same person despite their logical incompatibility. It is argued that the underlying distrust of food supply and chain systems in a local market rather than merely the consistency of the media content appears to motivate their acceptance.

It may be difficult to understand how people could believe the rumor that seaweed is made out of plastic or chickens used by the KFC fast food chain were genetically modified to have 6 wings and 8 legs. Nonetheless, food fraud involving some food category or with specific global brands (eg, KFC, Abbott, and Beingmate) has become an issue influencing conspiracy belief and causing economic loss. The rumors appeared in posts on WeChat, and the defamatory messages were read widely. The rumors are just some of many fake food stories going viral, where fears about the safety of food products have become deep-seated in the wake of major cases of food contamination, and food framed as artificial and dangerous may also function as a counterpoint to promote safe and sustainable food in China [46].

Weibo and WeChat were found to be the most used social media for accessing food information in China. For example, a video clip of news was circulated widely and had morphed into dozens of versions, accruing more than 2 million views on Weibo, China’s most popular social media platform. Nonetheless, there is an abundance of social media in China without gatekeepers for polarized communities within which antagonistic spheres are created, although they do foster engagement with food fraud discussion as well.

### Limitations

Although the HINTS-China provides evidence of media use in the persistence of conspiracy beliefs regarding food fraud rumors, it is almost impossible to disentangle the actual risks perceived from media exposure by researchers. Concomitantly, because we rely on self-reports of recall behavior, we cannot confirm that those reports reflect actual behavior. We also lack evidence to claim purposeful information-seeking behavior and the scanning use of health risk information on food fraud, since our study solely relied on a self-report method.

Another limitation that needs to be recognized is that it is not feasible to generalize the national probability sample to the Chinese population since the survey is only applicable to changes that occurred in 2017. The types of food fraud incidents in China are underrepresented. The effects of media use on food fraud conspiracy beliefs beyond that period remain to be studied. Finally, despite the ability to observe differences in conspiracy beliefs associated with media use in 2 cities, we cannot make strong causal claims because it still remains possible that characteristics other than those for which we controlled drove the changes in those beliefs.

### Conclusion

Passive media information seekers or those with information-scanning behavior were the majority who were exposed to information that was gathered incidentally from sources within their environment. Consequently, majority of respondents in Beijing and Hefei preferred to use interpersonal connection for learning food safety concerns, while very few respondents reported access to media run by public organizations for the same purpose. Nonetheless, the food fraud narratives examined according to the conspiracy belief showed that social media play important roles in influencing the attitude against negative reports, and 9 out of 11 food incidents tended to be perceived with higher conspiracy belief among Hefei respondents.

Food fraud is characterized with the intent to harm and is mainly done for economic gain. Thus, the main events of food fraud that occurred in 2016, specifically those associated with food rumors and incidents, were summarized in this study. The typical food fraud covered food adulteration, authenticity, contamination, crime, integrity, protection, quality, and safety, which were used as prompts for attitudinal and perceptual elicitation. Many food rumors place particular emphasis on compositional aspects of food, such as texture, color, and shape. They also involve food origin, geographical consideration, rearing or production systems, processing, and storage, among others. Food fraud regarding food originating from Japan, Vietnam, and New Zealand implies that the information concerning food fraud in these countries may negatively affect the valuation of import locations.

The food rumor narratives that examined the conspiracy belief of distrust degree showed that consumers with prior knowledge of food fraud incidents decreased the valuation of the sources less when they received further information about food fraud. Fueled by constant access to mobile devices, daily online media consumption has increased steadily since 2017. Chinese adults tend to spend more time each day listening to, reading, watching, or interacting with online media; however, their voracious appetite for digital content exists alongside a continued fondness for traditional media outlets. Furthermore, the results implied that prior consumer knowledge and later response to fraudulent behavior in a product can spread to other products.

## Appendix

Multimedia Appendix 1 Details of food fraud narratives and health risks of food incidents.

Multimedia Appendix 2 Media sources.

Multimedia Appendix 3 Sources for accessing food risk information.

Multimedia Appendix 4 Comparative analysis of the groups (low and high distrust) for learning about food rumors by accessing different media sources in Beijing and Hefei.

Multimedia Appendix 5 Comparative analysis of the groups (low and high distrust) for accessing media sources in learning about food in fraud and negative reports in Beijing and Hefei.

## Abbreviations

ANOVA: analysis of variance
CFDA: China Food & Drug Administration
GMF: genetically modified food
HINTS: Health Information National Trends Survey
HINTS-China: Health Information National Trends Survey in China
