# Supplementary material for: Media Source Characteristics Regarding Food Fraud Misinformation According to the Health Information National Trends Survey (HINTS) in China: Comparative Study
Source: JMIR Form Res. 2022 Mar 16;6(3):e32302. doi: 10.2196/32302 (PMC8968551; doi:10.2196/32302)
Supplement: Multimedia Appendix 1 [file formative_v6i3e32302_app1.docx]

**Multimedia Appendix 1.** Details of food fraud narratives and health risks of food incidents.

| Two official websites of the People's Republic of China verify the circulated rumors and misinformation: (人民网) <http://www.xinhuanet.com/english/> & Xinhuanet (新华网) <https://en.wikipedia.org/wiki/People%27s_Daily>.  **Textbox S1.** Conspiracy narratives of food rumors in 2017 HINTS-China survey (English translation in parenthesis).   \| **^1^ 有紫菜是由黑色塑膠片製作成的** \| \| --- \| \| (Edible seaweed (Nori) is made out of black plastic) \| \| **^2^ 又大又甜的草莓是打過藥的** \| \| (Big and sweet strawberries are made with excessive swelling agent) \| \| **^3^ 微波食品有害健康甚至會致癌** \| \| (Microwave food is harmful to health and causes cancer) \| \| **^4^ 方便麵是“垃圾食品”** \| \| (Instant noodles are "junk food.") \| \| **^5^ 小龍蝦基因是改造而來，用於處理屍體，且生長於污水中，重金屬超標** \| \| (Crayfish are genetically modified, deals with corpses, and grows in unsanitary water with exceeded levels of heavy metals pollutions) \| \| **^6^ 豬肉裡的鉤蟲“水煮不爛、高溫殺不死”** \| \| (Pork tapeworm come from eating under-cooked pork. Tapeworm in pork cannot be killed in water with high temperature. \| \| **^7^ 肯德基使用轉基因“六翅雞”** \| \| (KFC uses genetically modified chicken with six wings) \| \| **^8^ 無核的葡萄都是抹了避孕藥的** \| \| (Seedless grapes are spread with contraceptive medicine) \| |
| --- | --- | --- | --- | --- | --- | --- | --- | --- | --- | --- | --- | --- | --- | --- | --- | --- |

**Food Rumors in China (2016-2017)**

1. Nori is made out of black plastic.

<https://qz.com/934038/in-china-fake-news-about-food-goes-viral-because-people-find-it-hard-to-trust-anyone/>

Fact: The quality and safety of laver produced by the factories do not contain any plastic. It is a low-level common-sense mistake to confuse laver with plastic. Like any vegetable, seaweed have difference in taste and crispiness due to harvest time.

2. Big and sweet strawberry is grown with excess pesticide.

<https://www.webmd.com/food-recipes/news/20170309/ewg-dirty-dozen-pesticides>

Fact: Excessive pesticide can kill bees and other bugs, affects strawberry pollination, and reduces strawberry production. The strawberry dyeing process will reduce the storage and transportation duration. No farmer would take this risk.

3. Microwave food is harmful to health and causes cancer.

<https://www.cancer.net/blog/2021-03/can-using-microwave-cause-cancer>

Fact: The truth is that the main effect of the microwave oven is on the water molecules in the food, which will not have harmful effects on the human body.

4. Instant noodles are "junk food."

<http://kpzg.people.com.cn/n1/2016/0922/c404390-28733740.html>

Fact: There is no so-called junk food. The goal is to have balanced meals.

5. Crayfish are genetically modified, deals with corpses, and grows in unsanitary water with exceeded levels of heavy metals pollutions.

<http://shipin.people.com.cn/n1/2020/0706/c85914-31772242.html>

Fact: The survival rate of crayfish in a clean water environment can reach 80% or even 90%. Crayfish on the market are mostly farmed.

6. Pork tapeworm come from eating under-cooked pork. Tapeworm in pork cannot be killed in water with high temperature.

<http://shipin.people.com.cn/n1/2017/0111/c85914-29013877.html>

<http://shipin.people.com.cn/n/2015/0725/c85914-27360101.html>

Fact: there is no tapeworm in pork, and the pictures in pork are actually the blood vessels and lymphatic vessels.

7. KFC uses genetically modified chicken with six wings.

<http://www.xinhuanet.com/food/2017-04/25/c_1120871885.htm>

Fact: On May 26, 2015, KFC prosecuted 10 WeChat official accounts as the first batch of targets. The court judged that the three defendants were prominently located on the homepage of the news section of mainstream portals. The punishment was to apologize, and they were responsible for the plantiff’s compensation for the economic losses and reasonable rights protection costs of 600,000 yuan. Eventually, Xinmin.com, People.com, Sina.com and other media have successively refuted rumors, but with little success.

8. Seedless grapes are with contraceptives medication.

<http://jiaju.people.com.cn/n1/2017/0919/c151264-29544652.html>

Fact: Contraceptive are animal hormones and have no effect on plants.

| **Textbox S2.** Food incidents of food safety in 2016 (English translation in parenthesis) |
| --- |
| **^1^ 2016 年11 月底北京出現“活魚下架”事件，超市買不到活魚** |
| (The news covered the incidence of lacking fresh fish in the Beijing’s supermarkets. Therefore, there is no live fish available at the end of November 2016. Note: it was found later that it was merely the negative response of specific supermarkets to the strict supervision of food safety.) |
| **^2^ “餓了麼”網上訂餐被央視315 曝光黑作坊** |
| (An online food ordering and delivering app “Ele.me” was accused of partnering with unlicensed restaurants with poor sanitary conditions by the China Central Television (CCTV) 315 program) |
| **^3^ 漢麗軒被曝光牛排摻鴨肉** |
| (Beef steak at Hanlixuan restaurant containing duck meat is exposed) |
| **^4^ 北京簋街胡大等35 餐企使用罌粟殼被查** |
| (The use of poppy shells by 35 food enterprises such as Huda restaurant in Beijing Gui Street was banned) |
| **^5^ 上萬罐假冒雅培、貝因美等知名品牌的假奶粉流入市場** |
| (Thousands of cans of fake milk powder under the name of some famous brands such as Abbott and Beingmate entered the market) |
| **^6^ 日本輻射海鮮流入中國** |
| (Seafood from irradiated areas in Japan was smuggled into China) |
| **^7^ 百餘噸新西蘭過期乳製品流入上海，被重新包裝後賣出** |
| (More than 100 tons of expired milk powder from New Zealand was illegally repackaged and sold in Shanghai) |
| **^8^ 南方黑芝麻糊多次抽檢大腸菌群超標** |
| (The coliform of Nanfang Black Sesame exceeded the standard in a multiple sampling test) |
| **^9^ 無錫“楊銘宇”黃燜雞使用問題凍肉被曝光** |
| (Wuxi “Yang Mingyu” stewed chicken using problematic frozen meat was exposed) |
| **^10^ 越南三無優酪乳流入中國多地市場** |
| (Vietnam three-no yogurt entered the market in many cities in mainland China and was seized by CFDA) |
| **^11^ 國內23省44 城市自來水檢出疑似致癌物亞硝胺** |
| (Suspected carcinogen nitrosamines were detected in the tap water in 44 cities and in 23 provinces of China. Note: it was proved later inaccurate because the carcinogenic evidence of nitrosamine in human body is still unknown, and the low concentration of nitrosamine in tap water has little carcinogenic risk.) |

**Food incidents occurred and reported by the media (2016-2017).**

1. At the end of November 2016, there was a "live fish off the shelves" incident in Beijing, and supermarkets could not buy live fish

It is true: <https://view.news.qq.com/original/intouchtoday/n3724.html>

Event: In order to prevent live fish from detecting malachite green, the supermarket chose to take the initiative to remove it. It is just that the merchants passively respond to strict regulatory actions, but they have fermented into "food safety incidents."

2. "Are you hungry" online food ordering was exposed by CCTV 315 to the black workshop

It is true: <http://gz.people.com.cn/n2/2016/0316/c344102-27943842-2.html>

3. Han Lixuan was exposed to steak mixed with duck meat

It is true: <http://shipin.people.com.cn/n1/2017/0122/c85914-29041011.html>

4. 35 restaurant companies such as Hu Da, Guijie, Beijing, who used poppy shells were investigated

It is true: http://health.people.com.cn/n1/2016/0123/c14739-28078249.html

Event: Merchants are superstitious about the rumor that "poppy shells can improve flavor", believing that the addictive ingredients contained in poppy shells can make their home business prosperous. In fact, poppy husks are under the control of narcotic drugs, and the state explicitly prohibits the addition of poppy husks in food and cooking, which is an illegal and criminal act.

5. Thousands of cans of counterfeit milk powder from well-known brands such as Abbott and Beinmate enter the market

It is true: http://health.people.com.cn/n1/2016/1127/c408573-28899439.html

6. Japanese radioactive seafood flows into China

It is true: http://world.people.com.cn/n1/2016/0822/c1002-28655618.html

Event: The 230 million seafood smuggling case was solved, and some of the smuggled seafood was produced in the waters of Fukushima, Japan.

7. More than 100 tons of New Zealand expired dairy products flowed into Shanghai, repackaged, and sold

It is true: http://sn.people.com.cn/n2/2016/1025/c378297-29197719.html

Event: Case of illegal processing and sale of expired New Zealand imported bakery dairy products

8. The southern black sesame paste has been sampled and inspected for excessive coliform bacteria many times

It is true: http://shipin.people.com.cn/n1/2016/1006/c85914-28757576.html

9. Wuxi "Yang Mingyu" yellow chicken stew using frozen meat was exposed

It is true: http://health.people.com.cn/n1/2016/0420/c398004-28290488.html

10. Vietnam Sanwu yogurt flows into Chinese markets

It is true: http://shipin.people.com.cn/n1/2016/0407/c85914-28255941.html

11. Suspected carcinogen nitrosamines detected in tap water in 44 cities in 23 provinces in China

It is true: http://health.people.com.cn/n1/2016/1031/c21471-28820924.html

Event: The news was disseminated as a misinformation. Nitrosamines are category 2A carcinogens recognized by the World Health Organization, that is, a highly suspected carcinogen for which there is sufficient evidence in animal experiments, but the evidence of carcinogenesis in humans is not yet clear. However, the low concentration of nitrosamines in tap water has a low risk of cancer.
