# Supplementary material for: Media Source Characteristics Regarding Food Fraud Misinformation According to the Health Information National Trends Survey (HINTS) in China: Comparative Study
Source: JMIR Form Res. 2022 Mar 16;6(3):e32302. doi: 10.2196/32302 (PMC8968551; doi:10.2196/32302)
Supplement: Multimedia Appendix 2 [file formative_v6i3e32302_app2.docx]

**Multimedia Appendix 2.** Media sources.

**1. Interpersonal media**

1. Doctor or health specialist

2. Family member

3. Friend or colleague

**2. Traditional media**

4. Newspaper

5. Magazine

6. Television

7. Radio

8. Book

16. Telephone hotline

**3. Public organizations (government and non-profit organizations)**

9. Official government agency

10. International organization

11. Academic research institution

12. Business organization

13. Religious organization or leader

14. Community or neighborhood committee

15. Charitable organization

**4. Internet portal**

17. Website

18. News APP

19. Professional APP

20. Other APP, besides abovementioned APP

21. Search engine

**5. Social media**

22. MicroBlog

23. WeChat

24. Blog and forum
