# Supplementary material for: Media Source Characteristics Regarding Food Fraud Misinformation According to the Health Information National Trends Survey (HINTS) in China: Comparative Study
Source: JMIR Form Res. 2022 Mar 16;6(3):e32302. doi: 10.2196/32302 (PMC8968551; doi:10.2196/32302)
Supplement: Multimedia Appendix 3 [file formative_v6i3e32302_app3.docx]

**Multimedia Appendix 3.** Sources for accessing food risk information.

**Source for accessing food risk information in Beijing**

|  | 1^st^  N (%) | 2^nd^  N (%) | 3^rd^  N (%) |
| --- | --- | --- | --- |
| 1. Doctor or health specialist | 264(72.9) | 50(13.8) | 48(13.3) |
| 2. Family member | 73(22.5) | 197(60.8) | 54(16.7) |
| 6. Television | 51(31.5) | 54(33.3) | 57(35.2) |
| 3. Friend or colleague | 48(16.4) | 84(28.7) | 161(54.9) |
| 17. Website | 41(36.6) | 27(24.1) | 44(39.3) |
| 9. Official government agency | 28(27.7) | 35(34.7) | 38(37.6) |
| 19. Professional APP | 12(37.5) | 10(31.3) | 10(31.3) |
| 8. Book | 9(20.9) | 17(39.5) | 17(39.5) |
| 23. WeChat | 8(16.7) | 11(22.9) | 29(60.4) |
| 18. News APP | 7(20) | 17(48.6) | 11(31.4) |
| 21. Search engine | 7(16.3) | 9(20.9) | 27(62.8) |
| 5. Magazine | 6(24) | 12(48) | 7(28) |
| 4. Newspaper | 5(15.2) | 14(42.4) | 14(42.4) |
| 11. Academic research institution | 3(12) | 6(24) | 16(64) |
| 7. Radio | 3(13.6) | 9(40.9) | 10(45.5) |
| 20. Other APP | 2(20) | 2(20) | 6(60) |
| 13. Religious organization or leader | 1(25) | 2(50) | 1(25) |
| 22. Micro Blog | 1(9.1) | 6(54.5) | 4(36.4) |
| 16. Telephone hotline | 1(11.1) | 5(55.6) | 3(33.3) |
| 10. International organization | 0(0) | 0(0) | 3(100) |
| 12. Business organization | 0(0) | 2(40) | 3(60) |
| 14. Community or neighborhood committee | 0(0) | 1(14.3) | 6(85.7) |
| 15. Charitable organization | 0(0) | 0(0) | 0(0) |
| 24. Blog and forum | 0(0) | 0(0) | 1(100) |
| 25. Others | 0(0) | 0(0) | 0(0) |

**Source for accessing food risk information in Hefei**

|  | 1st  N (%) | 2nd  N(%) | 3rd  N (%) |
| --- | --- | --- | --- |
| 1. Doctor or health specialist | 112(74.2) | 17(11.3) | 22(14.6) |
| 17. Website | 53(43.4) | 27(22.1) | 42(34.4) |
| 2. Family member | 52(30.8) | 85(50.3) | 32(18.9) |
| 3. Friend or colleague | 45(26.9) | 53(31.7) | 69(41.3) |
| 6. Television | 31(28.7) | 32(29.6) | 45(41.7) |
| 21. Search engine | 25(55.6) | 17(37.8) | 3(6.7) |
| 4. Newspaper | 15(34.9) | 16(3.2) | 12(27.9) |
| 18. News APP | 13(37.1) | 19(54.3) | 3(8.6) |
| 23. WeChat | 8(13.1) | 17(27.9) | 36(59) |
| 7. Radio | 7(19.4) | 13(36.1) | 16(44.4) |
| 8. Book | 7(14.6) | 20(41.7) | 21(43.8) |
| 19. Professional APP | 6(33.30 | 4(22.2) | 8(44.4) |
| 16. Telephone hotline | 6(33.3) | 2(11.1) | 10(55.6) |
| 9. Official government agency | 6(31.6) | 7(36.8) | 6(31.6) |
| 12. Business organization | 3(16.7) | 8(44.4) | 7(38.9) |
| 5. Magazine | 3(15) | 9(45) | 8(40) |
| 14. Community or neighborhood committee | 1(11.1) | 1(11.1) | 7(77.8) |
| 10. International organization | 0(0) | 4(80) | 1(20) |
| 11. Academic research institution | 0(0) | 8(34.8) | 15(65.2) |
| 13. Religious organization or leader | 0(0) | 0(0) | 2(100) |
| 15. Charitable organization | 0(0) | 1(33.3) | 2(66.7) |
| 20. Other APP | 0(0) | 1(16.7) | 5(83.3) |
| 22. Micro Blog | 0(0) | 26(78.8) | 7(21.2) |
| 24. Blog and forum | 0(0) | 0(0) | 4(100) |
| 25. Others | 0(0) | 0(0) | 4(100) |
