# Supplementary material for: Media Source Characteristics Regarding Food Fraud Misinformation According to the Health Information National Trends Survey (HINTS) in China: Comparative Study
Source: JMIR Form Res. 2022 Mar 16;6(3):e32302. doi: 10.2196/32302 (PMC8968551; doi:10.2196/32302)
Supplement: Multimedia Appendix 4 [file formative_v6i3e32302_app4.docx]

**Multimedia Appendix 4.** Comparative analysis of the groups (low and high distrust) for learning about food rumors by accessing different media sources in Beijing and Hefei.

| Conspiracy narratives | Beijing | | | | Hefei | | | |
| --- | --- | --- | --- | --- | --- | --- | --- | --- |
| Plastic seaweed | Total 727(%) | low  502(%) | High 225(%) | χ^2^_4_ | Total 310(%) | Incidental  253(%) | Active 57(%) | χ^2^_4_ |
| Interpersonal connection | 229(31.5) | 183(36.5) | 46(20.4) | 29.26*** | 85(27.4) | 65(25.7) | 20(35.1) | 17.66** |
| Public organizations | 1(0.1) | 1(0.2) | 0(0.0) |  | 3(1.0) | 2(0.8) | 1(1.8) |  |
| Traditional media | 199(27.4) | 113(22.5) | 86(38.2) |  | 91(29.4) | 74(29.2) | 17(29.8) |  |
| Internet portal | 132(18.2) | 85(16.9) | 47(20.9) |  | 71(22.9) | 57(22.5) | 14(24.6) |  |
| Social media | 166(22.8) | 120(23.9) | 46(20.4) |  | 58(18.7) | 55(21.7) | 3(5.3) |  |
| Others | 0(0.0) | 0(0.0) | 0(0.0) |  | 2(0.6) | 0(0.0) | 2(3.5) |  |
| Additive  Strawberries | 803(%) | 775(%) | 28(%) | χ^2^_5_ | 547(%) | 235(%) | 312(%) | χ^2^_5_ |
| Interpersonal connection | 475(59.2) | 466(60.1) | 9(32.1) | 11.00 | 291(53.2) | 127(54.0) | 164(52.6) | 6.79 |
| Public organizations | 8(1.0) | 8(1.0) | 0(0.0) |  | 3(0.5) | 3(1.3) | 0(0.0) |  |
| Traditional media | 128(15.9) | 120(15.5) | 8(28.6) |  | 154(28.2) | 68(28.9) | 86(27.6) |  |
| Internet portal | 97(12.1) | 91(11.7) | 6(21.4) |  | 66(12.1) | 27(11.5) | 39(12.5) |  |
| Social media | 82(10.2) | 77(9.9) | 5(17.9) |  | 32(5.9) | 10(4.3) | 22(7.1) |  |
| Others | 13(1.6) | 13(1.7) | 0(0.0) |  | 1(0.2) | 0(0.0) | 1(0.3) |  |
| Microwave food | 716(%) | 646(%) | 70(%) | χ^2^_5_ | 314(%) | 279(%) | 35(%) | χ^2^_5_ |
| Interpersonal connection | 356(49.7) | 323(50.0) | 33(47.1) | .37 | 156(49.7) | 141(50.5) | 15(42.9) | 4.34 |
| Public organizations | 10(1.4) | 9(1.4) | 1(1.4) |  | 2(0.6) | 2(0.7) | 0(0.0) |  |
| Traditional media | 193(27.0) | 173(26.8) | 20(28.6) |  | 85(27.1) | 77(27.6) | 8(22.9) |  |
| Internet portal | 120(16.8) | 108(16.7) | 12(17.1) |  | 46(14.6) | 37(13.3) | 9(25.7) |  |
| Social media | 36(5.0) | 32(5.0) | 4(5.7) |  | 24(7.6) | 21(7.5) | 3(8.6) |  |
| Others | 1(0.1) | 1(0.2) | 0(0.0) |  | 1(0.3) | 1(0.4) | 0(0.0) |  |
| ^C5.4^ Instant noodles | 1253(%) | 978(%) | 275(%) | χ^2^_5_ | 946(%) | 600(%) | 346(%) | χ^2^_5_ |
| Interpersonal connection | 698(55.7) | 568(58.1) | 130(47.3) | 20.08** | 534(56.4) | 352(58.7) | 182(52.6) | 8.74 |
| Public organizations | 12(1.0) | 10(1.0) | 2(0.7) |  | 8(0.8) | 5(0.8) | 3(0.9) |  |
| Traditional media | 325(25.9) | 253(25.9) | 72(26.2) |  | 280(29.6) | 167(27.8) | 113(32.7) |  |
| Internet portal | 144(11.5) | 95(9.7) | 49(17.8) |  | 82(8.7) | 51(8.5) | 31(9.0) |  |
| Social media | 72(5.7) | 51(5.2) | 21(7.6) |  | 32(3.4) | 16(2.7) | 16(4.6) |  |
| Others | 2(0.2) | 1(0.1) | 1(0.4) |  | 10(1.1) | 9(1.5) | 1(0.3) |  |
| ^C5.5^ Crayfish | 436(%) | 381(%) | 55(%) | χ^2^_5_ | 296(%) | 266(%) | 30(%) | χ^2^_5_ |
| Interpersonal connection | 161(36.9) | 149(39.1) | 12(21.8) | 29.87*** | 137(46.3) | 123(46.2) | 14(46.7) | 2.31 |
| Public organizations | 5(1.1) | 4(1.0) | 1(1.8) |  | 3(1.0) | 3(1.1) | 0(0.0) |  |
| Traditional media | 126(28.9) | 119(31.2) | 7(12.7) |  | 88(29.7) | 80(30.1) | 8(26.7) |  |
| Internet portal | 87(20.0) | 64(16.8) | 23(41.8) |  | 39(13.2) | 36(13.5) | 3(10.0) |  |
| Social media | 54(12.4) | 42(11.0) | 12(21.8) |  | 23(7.8) | 19(7.1) | 4(13.3) |  |
| Others | 3(0.7) | 3(0.8) | 0(0.0) |  | 6(2.0) | 5(1.9) | 1(3.3) |  |
| ^C5.6^ Hookworm in pork | 481(%) | 426(%) | 55(%) | χ^2^_5_ | 253(%) | 216(%) | 37(%) | χ^2^_5_ |
| Interpersonal connection | 188(39.1) | 176(41.3) | 12(21.8) | 40.18*** | 92(36.4) | 79(36.6) | 13(35.1) | 5.49 |
| Public organizations | 11(2.3) | 11(2.6) | 0(0.0) |  | 1(0.4) | 1(0.5) | 0(0.0) |  |
| Traditional media | 136(28.3) | 129(30.3) | 7(12.7) |  | 85(33.6) | 71(32.9) | 14(37.8) |  |
| Internet portal | 63(13.1) | 51(12.0) | 12(21.8) |  | 45(17.8) | 39(18.1) | 6(16.2) |  |
| Social media | 80(16.6) | 57(13.4) | 23(41.8) |  | 26(10.3) | 24(11.1) | 2(5.4) |  |
| Others | 3(0.6) | 2(0.5) | 1(1.8) |  | 4(1.6) | 2(0.9) | 2(5.4) |  |
| ^C5.7^ Six wing chicken | 794(%) | 700(%) | 94(%) | χ^2^_4_ | 449(%) | 182(%) | 267(%) | χ^2^_5_ |
| Interpersonal connection | 230(29.0) | 206(29.4) | 24(25.5) | 3.89 | 157(35.0) | 65(35.7) | 92(34.5) | 1.36 |
| Public organizations | 5(0.6) | 5(0.7) | 0(0.0) |  | 3(0.7) | 1(0.5) | 2(0.7) |  |
| Traditional media | 228(28.7) | 205(29.3) | 23(24.5) |  | 149(33.2) | 63(34.6) | 86(32.2) |  |
| Internet portal | 178(22.4) | 151(21.6) | 27(28.7) |  | 103(22.9) | 37(20.3) | 66(24.7) |  |
| Social media | 153(19.3) | 133(19.0) | 20(21.3) |  | 35(7.8) | 15(8.2) | 20(7.5) |  |
| Others | 0(0.0) | 0(0.0) | 0(0.0) |  | 2(0.4) | 1(0.5) | 1(0.4) |  |
| ^C5.8^ Seedless grapes | 346(%) | 302(%) | 44(%) | χ^2^_5_ | 155(%) | 138(%) | 17(%) | χ^2^_4_ |
| Interpersonal connection | 122(35.3) | 116(38.4) | 6(13.6) | 19.95** | 61(39.4) | 52(37.7) | 9(52.9) | 1.77 |
| Public organizations | 7(2.0) | 4(1.3) | 3(6.8) |  | 1(0.6) | 1(0.7) | 0(0.0) |  |
| Traditional media | 79(22.8) | 62(20.5) | 17(38.6) |  | 49(31.6) | 45(32.6) | 4(23.5) |  |
| Internet portal | 64(18.5) | 53(17.5) | 11(25.0) |  | 17(11.0) | 15(10.9) | 2(11.8) |  |
| Social media | 72(20.8) | 65(21.5) | 7(15.9) |  | 27(17.4) | 25(18.1) | 2(11.8) |  |
| Others | 2(0.6) | 2(0.7) | 0(0.0) |  | 0(0.0) | 0(0.0) | 0(0.0) |  |
| Note: ***P* < .01, ****P* < .001 | | | | | | | | |
