# Supplementary material for: Media Source Characteristics Regarding Food Fraud Misinformation According to the Health Information National Trends Survey (HINTS) in China: Comparative Study
Source: JMIR Form Res. 2022 Mar 16;6(3):e32302. doi: 10.2196/32302 (PMC8968551; doi:10.2196/32302)
Supplement: Multimedia Appendix 5 [file formative_v6i3e32302_app5.docx]

**Multimedia Appendix 5.** Comparative analysis of the groups (low and high distrust) for accessing media sources in learning about food in fraud and negative reports in Beijing and Hefei.

| Food scandals | Beijing | | | | Hefei | | | |
| --- | --- | --- | --- | --- | --- | --- | --- | --- |
| ^C6.1^ Live fish off the shelves | Total 1462(%) | Low 617(%) | High 845(%) | χ^2^_5_ | Total 1122(%) | Low 324(%) | High 798(%) | χ^2^_5_ |
| Interpersonal connection | 362(24.8) | 279(45.2) | 83(9.8) | 636.33*** | 208(18.5) | 94(29.0) | 114(14.3) | 192.86*** |
| Public organizations | 22(1.5) | 16(2.6) | 6(0.7) |  | 15(1.3) | 7(2.2) | 8(1.0) |  |
| Traditional media | 225(15.4) | 175(28.4) | 50(5.9) |  | 330(29.4) | 145(44.8) | 185(23.2) |  |
| Internet portal | 106(7.3) | 68(11.0) | 38(4.5) |  | 138(12.3) | 46(14.2) | 92(11.5) |  |
| Social media | 503(34.4) | 65(10.5) | 438(51.8) |  | 50(4.5) | 20(6.2) | 30(3.8) |  |
| Others | 244(16.7) | 14(2.3) | 230(27.2) |  | 381(34.0) | 12(3.7) | 369(46.2) |  |
| ^C6.2^ "Ele.me" online ordering | Total 1462(%) | Low 857(%) | High 605(%) | χ^2^_5_ | Total 1122(%) | Low 296(%) | High 826(%) | χ^2^_5_ |
| Interpersonal connection | 210(14.4) | 180(21.0) | 30(5.0) | 932.82*** | 197(17.6) | 44(14.9) | 153(18.5) | 148.07*** |
| Public organizations | 8(0.5) | 5(0.6) | 3(0.5) |  | 12(1.1) | 8(2.7) | 4(0.5) |  |
| Traditional media | 557(38.1) | 494(57.6) | 63(10.4) |  | 454(40.5) | 179(60.5) | 275(33.3) |  |
| Internet portal | 158(10.8) | 128(14.9) | 30(5.0) |  | 137(12.2) | 47(15.9) | 90(10.9) |  |
| Social media | 69(4.7) | 45(5.3) | 24(4.0) |  | 58(5.2) | 17(5.7) | 41(5.0) |  |
| Others | 460(31.5) | 5(0.6) | 455(75.2) |  | 264(23.5) | 1(0.3) | 263(31.8) |  |
| ^C6.3^ Hanlixuan restaurant | Total 1462(%) | Low 590(%) | High 872(%) | χ^2^_5_ | Total 1122(%) | Low 358(%) | High 764(%) | χ^2^_5_ |
| Interpersonal connection | 237(16.2) | 191(32.4) | 46(5.3) | 922.32*** | 195(17.4) | 91(25.4) | 104(13.6) | 230.69*** |
| Public organizations | 7(0.5) | 1(0.2) | 6(0.7) |  | 10(0.9) | 7(2.0) | 3(0.4) |  |
| Traditional media | 288(19.7) | 235(39.8) | 53(6.1) |  | 341(30.4) | 156(43.6) | 185(24.2) |  |
| Internet portal | 148(10.1) | 110(18.6) | 38(4.4) |  | 155(13.8) | 72(20.1) | 83(10.9) |  |
| Social media | 80(5.5) | 51(8.6) | 29(3.3) |  | 56(5.0) | 26(7.3) | 30(3.9) |  |
| Others | 702(48.0) | 2(0.3) | 700(80.3) |  | 365(32.5) | 6(1.7) | 359(47.0) |  |
| ^C6.4^ Use of poppy shells | Total 1462(%) | Low 615(%) | High 847(%) | χ^2^_5_ | Total 1122(%) | Low 274(%) | High 848(%) | χ^2^_5_ |
| Interpersonal connection | 341(23.3) | 290(47.2) | 51(6.0) | 905.66*** | 180(16.0) | 66(24.1) | 114(13.4) | 184.53*** |
| Public organizations | 15(1.0) | 9(1.5) | 6(0.7) |  | 15(1.3) | 7(2.6) | 8(0.9) |  |
| Traditional media | 231(15.8) | 194(31.5) | 37(4.4) |  | 314(28.0) | 127(46.4) | 187(22.1) |  |
| Internet portal | 107(7.3) | 74(12.0) | 33(3.9) |  | 163(14.5) | 57(20.8) | 106(12.5) |  |
| Social media | 146(10.0) | 44(7.2) | 102(12.0) |  | 48(4.3) | 10(3.6) | 38(4.5) |  |
| Others | 622(42.5) | 4(0.7) | 618(73.0) |  | 402(35.8) | 7(2.6) | 395(46.6) |  |
| ^C6.5^ Fake milk powder | Total 1462(%) | Low 548(%) | High 914(%) | χ^2^_5_ | Total 1122(%) | Low 538(%) | High 584(%) | χ^2^_5_ |
| Interpersonal connection | 219(15.0) | 170(31.0) | 49(5.4) | 834.79*** | 194(17.3) | 113(21.0) | 81(13.9) | 293.47*** |
| Public organizations | 30(2.1) | 22(4.0) | 8(0.9) |  | 34(3.0) | 23(4.3) | 11(1.9) |  |
| Traditional media | 285(19.5) | 236(43.1) | 49(5.4) |  | 387(34.5) | 250(46.5) | 137(23.5) |  |
| Internet portal | 143(9.8) | 87(15.9) | 56(6.1) |  | 186(16.6) | 113(21.0) | 73(12.5) |  |
| Social media | 224(15.3) | 32(5.8) | 192(21.0) |  | 59(5.3) | 34(6.3) | 25(4.3) |  |
| Others | 561(38.4) | 1(0.2) | 560(61.3) |  | 262(23.4) | 5(0.9) | 257(44.0) |  |
| ^C6.6^ Radiation of seafood | Total 1462(%) | Low 675(%) | High 787(%) | χ^2^_5_ | Total 1122(%) | Low 557(%) | High 565(%) | χ^2^_5_ |
| Interpersonal connection | 204(14.0) | 171(25.3) | 33(4.2) | 863.82*** | 176(15.7) | 106(19.0) | 70(12.4) | 320.75*** |
| Public organizations | 44(3.0) | 40(5.9) | 4(0.5) |  | 36(3.2) | 26(4.7) | 10(1.8) |  |
| Traditional media | 294(20.1) | 250(37.0) | 44(5.6) |  | 378(33.7) | 250(44.9) | 128(22.7) |  |
| Internet portal | 176(12.0) | 144(21.3) | 32(4.1) |  | 192(17.1) | 124(22.3) | 68(12.0) |  |
| Social media | 269(18.4) | 66(9.8) | 203(25.8) |  | 68(6.1) | 44(7.9) | 24(4.2) |  |
| Others | 475(32.5) | 4(0.6) | 471(59.8) |  | 272(24.2) | 7(1.3) | 265(46.9) |  |
| ^C6.7^ New Zealand expired dairy products | Total 1462(%) | Low 484(%) | High 978(%) | χ^2^_5_ | Total 1122(%) | Low 351(%) | High 771(%) | χ^2^_5_ |
| Interpersonal connection | 206(14.1) | 176(36.4) | 30(3.1) | 877.23*** | 191(17.0) | 84(23.9) | 107(13.9) | 219.60*** |
| Public organizations | 26(1.8) | 18(3.7) | 8(0.8) |  | 22(2.0) | 11(3.1) | 11(1.4) |  |
| Traditional media | 208(14.2) | 166(34.3) | 42(4.3) |  | 306(27.3) | 150(42.7) | 156(20.2) |  |
| Internet portal | 132(9.0) | 85(17.6) | 47(4.8) |  | 197(17.6) | 83(23.6) | 114(14.8) |  |
| Social media | 308(21.1) | 37(7.6) | 271(27.7) |  | 54(4.8) | 18(5.1) | 36(4.7) |  |
| Others | 582(39.8) | 2(0.4) | 580(59.3) |  | 352(31.4) | 5(1.4) | 347(45.0) |  |
| ^C6.8^ Nanfang Black Sesame | Total 1462(%) | Low 553(%) | High 909(%) | χ^2^_5_ | Total 1122(%) | Low 329(%) | High 793(%) | χ^2^_5_ |
| Interpersonal connection | 232(15.9) | 205(37.1) | 27(3.0) | 913.15*** | 190(16.9) | 80(24.3) | 110(13.9) | 215.52*** |
| Public organizations | 14(1.0) | 10(1.8) | 4(0.4) |  | 20(1.8) | 11(3.3) | 9(1.1) |  |
| Traditional media | 237(16.2) | 195(35.3) | 42(4.6) |  | 312(27.8) | 149(45.3) | 163(20.6) |  |
| Internet portal | 130(8.9) | 94(17.0) | 36(4.0) |  | 167(14.9) | 61(18.5) | 106(13.4) |  |
| Social media | 306(20.9) | 47(8.5) | 259(28.5) |  | 67(6.0) | 23(7.0) | 44(5.5) |  |
| Others | 543(37.1) | 2(0.4) | 541(59.5) |  | 366(32.6) | 5(1.5) | 361(45.5) |  |
| ^C6.9^ Wuxi "Yang Mingyu" | Total 1462(%) | Low 515(%) | High 947(%) | χ^2^_5_ | Total 1122(%) | Low 421(%) | High 701(%) | χ^2^_5_ |
| Interpersonal connection | 225(15.4) | 181(35.1) | 44(4.6) | 847.99*** | 222(19.8) | 116(27.6) | 106(15.1) | 256.57*** |
| Public organizations | 11(0.8) | 8(1.6) | 3(0.3) |  | 23(2.0) | 8(1.9) | 15(2.1) |  |
| Traditional media | 228(15.6) | 186(36.1) | 42(4.4) |  | 320(28.5) | 164(39.0) | 156(22.3) |  |
| Internet portal | 105(7.2) | 80(15.5) | 25(2.6) |  | 175(15.6) | 91(21.6) | 84(12.0) |  |
| Social media | 312(21.3) | 58(11.3) | 254(26.8) |  | 72(6.4) | 41(9.7) | 31(4.4) |  |
| Others | 581(39.7) | 2(0.4) | 579(61.1) |  | 310(27.6) | 1(0.2) | 309(44.1) |  |
| ^C6.10^ Vietnam three-no yogurt | Total 1462(%) | Low 442(%) | High 1020(%) | χ^2^_5_ | Total 1122(%) | Low 339(%) | High 783(%) | χ^2^_5_ |
| Interpersonal connection | 191(13.1) | 156(35.3) | 35(3.4) | 839.82*** | 196(17.5) | 88(26.0) | 108(13.8) | 216.42*** |
| Public organizations | 9(0.6) | 5(1.1) | 4(0.4) |  | 27(2.4) | 15(4.4) | 12(1.5) |  |
| Traditional media | 208(14.2) | 159(36.0) | 49(4.8) |  | 298(26.6) | 128(37.8) | 170(21.7) |  |
| Internet portal | 129(8.8) | 84(19.0) | 45(4.4) |  | 174(15.5) | 80(23.6) | 94(12.0) |  |
| Social media | 316(21.6) | 37(8.4) | 279(27.4) |  | 53(4.7) | 21(6.2) | 32(4.1) |  |
| Others | 609(41.7) | 1(0.2) | 608(59.6) |  | 374(33.3) | 7(2.1) | 367(46.9) |  |
| ^C6.11^ Carcinogen in tap water | Total 1462(%) | Low 471(%) | High 991(%) | χ^2^_5_ | Total 1122(%) | Low 359(%) | High 763(%) | χ^2^_5_ |
| Interpersonal connection | 184(12.6) | 140(29.7) | 44(4.4) | 862.86*** | 213(19.0) | 88(24.5) | 125(16.4) | 231.06*** |
| Public organizations | 29(2.0) | 27(5.7) | 2(0.2) |  | 30(2.7) | 19(5.3) | 11(1.4) |  |
| Traditional media | 228(15.6) | 183(38.9) | 45(4.5) |  | 314(28.0) | 163(45.4) | 151(19.8) |  |
| Internet portal | 112(7.7) | 79(16.8) | 33(3.3) |  | 155(13.8) | 63(17.5) | 92(12.1) |  |
| Social media | 285(19.5) | 41(8.7) | 244(24.6) |  | 51(4.5) | 18(5.0) | 33(4.3) |  |
| Others | 624(42.7) | 1(0.2) | 623(62.9) |  | 359(32.0) | 8(2.2) | 351(46.0) |  |
| Note: ****P* < .001 | | | | | | | | |
